# Cutting-edge approaches for targeted drug delivery in breast cancer: beyond conventional therapies

**DOI:** 10.1039/d4na00086b

**Published:** 2024-04-10

**Authors:** Ramesh Chaudhari, Vishva Patel, Ashutosh Kumar

**Affiliations:** a Biological & Life Sciences, School of Arts & Sciences, Ahmedabad University Central Campus, Navrangpura Ahmedabad 380009 Gujarat India ashutosh.kumar@ahduni.edu.in

## Abstract

Breast cancer is a global health challenge with staggering statistics underscoring its pervasive impact. The burden of this disease is measured in terms of its prevalence and the challenges it poses to healthcare systems, necessitating a closer look at its epidemiology and impact. Current breast cancer treatments, including surgery, chemotherapy, radiation therapy, and targeted therapies, have made significant strides in improving patient outcomes. However, they are not without limitations, often leading to adverse effects and the development of drug resistance. This comprehensive review delves into the complex landscape of breast cancer, including its incidence, current treatment modalities, and the inherent limitations of existing therapeutic approaches. It also sheds light on the promising role of nanotechnology, encompassing both inorganic and organic nanoparticles equipped with the ability to selectively deliver therapeutic agents to tumor sites, in the battle against breast cancer. The review also addresses the emerging therapies, their associated challenges, and the future prospects of targeted drug delivery in breast cancer management.

## Abstract 2

Discover the groundbreaking potential of nanotech-based targeted delivery in breast cancer therapy. Our review showcases how this innovative precision medicine approach can revolutionise treatment and improve patient outcomes.

## Graphical abstract

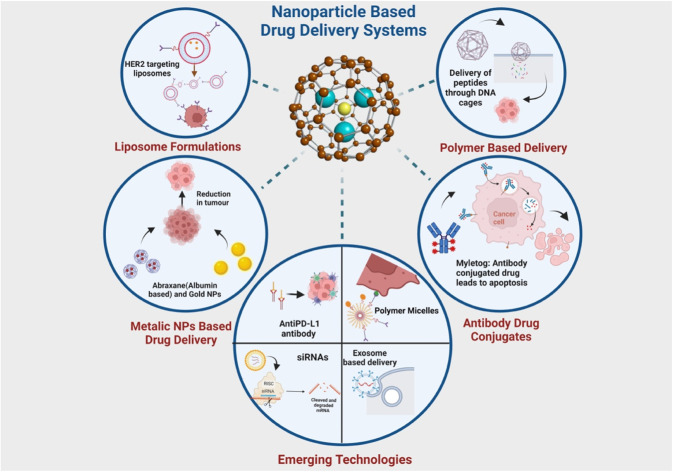

## 1. Introduction

Cancer, a complex and devastating group of diseases characterised by uncontrolled cell growth and tissue invasion, poses a formidable challenge to healthcare systems worldwide.^1,2^ Among the various cancer types, breast cancer is one of the most prevalent and studied malignancies, exhibiting diverse subtypes based on molecular characteristics.^3,4^ In 2022, the mortality rate for breast cancer among diagnosed women amounted to approximately 29.1%, with 670 000 fatalities reported out of 2.3 million cases.^5^ Although advancements in early detection and treatment have improved patient outcomes, breast cancer remains a significant global health concern.^6^ Challenges such as late-stage diagnosis, genetic predisposition, and limited healthcare access in underserved regions persist.^7,8^

Breast cancer is multifaceted, and understanding its molecular heterogeneity is crucial for tailored treatment strategies.^9^ Recent studies have illuminated the molecular intricacies of breast cancer, revealing distinct subtypes with unique clinical behaviours and therapeutic responses.^10^ Identifying molecular markers, such as hormone receptors and Human epidermal growth factor receptor 2 (HER2) status, has revolutionised treatment approaches, enabling targeted therapies like Herceptin for HER2-positive breast cancer.^11,12^ However, despite these advances, late-stage diagnosis remains a significant concern. Efforts have been made to improve early detection through screening programs and developing advanced imaging techniques.^13^ As demonstrated by BRCA1 and BRCA2 mutations, genetic predisposition also plays a critical role in breast cancer risk assessment and prevention.^14^

**Fig. 1:**
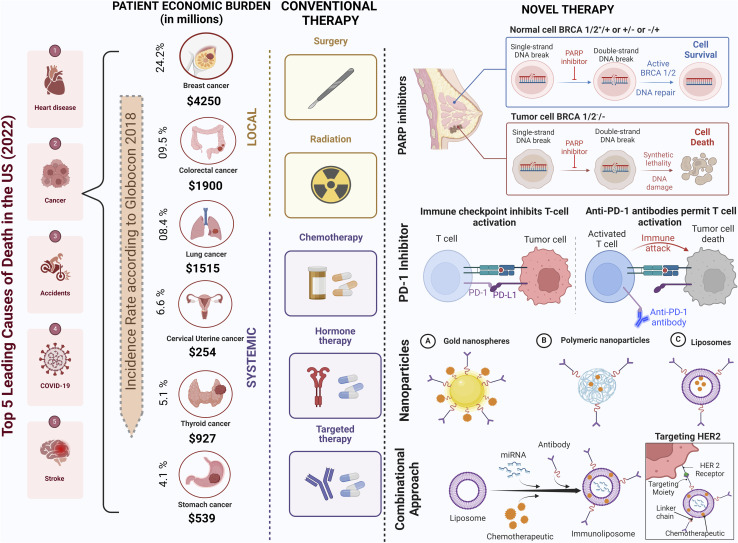
Global burden of cancer and progress in targeted therapies. Cancer constitutes a major worldwide health burden as a leading cause of mortality and economic costs. Breast cancer is the most prevalent cancer in women. While conventional therapies like chemotherapy, radiation and surgery underpin treatment, they have limitations in selectivity and toxicity. Recent advances such as PD-1 and PARP inhibitors, nanoparticle drug delivery systems, and combination targeted therapies demonstrate the potential to improve therapeutic outcomes through precision delivery and mechanisms targeting cancer cell vulnerabilities. This figure has been created with https://www.biorender.com, copyright 2024.

Current treatment approaches encompass a multidisciplinary approach, including surgery (lumpectomy and mastectomy), radiation therapy, chemotherapy, and hormonal therapy, depending on the tumor type and stage.^15^ While these modalities have made substantial strides in improving patient outcomes, they are not without drawbacks.^16^ Chemotherapy and radiation therapy frequently result in debilitating side effects such as nausea and fatigue, while surgical interventions may carry risks of organ damage and postoperative complications.^17^ Moreover, traditional therapies may exhibit limited efficacy, particularly in advanced or metastatic disease, and can inadvertently foster resistance in cancer cells. Furthermore, their invasive nature and high financial costs can significantly impact patients' well-being and financial stability.^18^ Nevertheless, traditional therapies remain indispensable components of cancer care, often synergizing with emerging targeted therapies and immunotherapies to optimize treatment outcomes. For instance, adjuvant radiation treatment has dramatically increased survival rates and decreased the chance of recurrence.^19^ In recent years, immunotherapy has emerged as a promising avenue for breast cancer treatment. Immune checkpoint inhibitors, such as programmed cell death protein 1 (PD-1) and programmed cell death ligand 1 (PD-L1) inhibitors, are being investigated in clinical trials, harnessing the immune system to combat cancer cells (Fig. 1).^20^ Guided by genomics and biomarker research, personalised medicine allows for customised treatment plans based on an individual's unique cancer profile, optimising therapeutic outcomes.^21^

Targeted delivery systems are indispensable in cancer therapy because they enhance drug efficacy by precisely delivering therapeutics to tumor sites, thereby increasing drug concentration at the target tissue while minimising off-target effects.^22^ Moreover, these systems facilitate the circumvention of biological barriers like the blood–brain barrier, enabling effective drug delivery to otherwise inaccessible regions. Additionally, personalised medicine approaches made possible by targeted delivery systems offer tailored treatments, optimising therapeutic outcomes and reducing adverse reactions in cancer patients.^23^ Furthermore, targeted drug delivery systems, a vital focus of this review, have garnered significant attention in breast cancer therapy. Nanotechnology-based delivery systems, such as liposomes and nanoparticles, can enhance drug bioavailability, reduce systemic toxicity, and improve drug stability.^24,25^

## 2. Significance of targeted drug delivery in breast cancer

The complexity of breast cancer surpasses that of many other cancers due to various factors. Research indicates that breast cancer exhibits considerable heterogeneity, with distinct molecular subtypes, such as luminal A, luminal B, HER2-enriched, and triple-negative breast cancer (TNBC), each having different biological behaviours and responses to treatment.^3,10^ This heterogeneity poses challenges in developing effective treatment strategies tailored to individual patients. Targeted drug delivery in breast cancer is a transformative approach with significant implications for improving treatment outcomes.^26^ This approach is particularly significant as it addresses the challenges associated with conventional chemotherapy and radiation therapy, which often result in systemic toxicity and severe side effects.^27^ Advancements in nanotechnology have enabled innovative drug delivery systems, such as nanoparticles and liposomes, that can encapsulate chemotherapy drugs or targeted agents and selectively release them within the tumor microenvironment.^28^ These nanocarriers can improve drug bioavailability, enhance drug stability, and prolong drug circulation times, thereby increasing drug concentration at the tumor site and minimising off-target effects.^29^

Furthermore, the emergence of targeted therapies, such as monoclonal antibodies and small molecule inhibitors, has revolutionised breast cancer treatment.^12^ In the past two decades, two HER2-targeted monoclonal antibodies (mAbs), trastuzumab and pertuzumab, have been approved as adjuvant treatments for HER2^+^ breast cancer and metastatic breast cancer. Trastuzumab, an early breakthrough in targeted oncology therapy, serves as the cornerstone for HER2^+^ breast cancer treatment. Trastuzumab acts through various mechanisms, including the blocking of downstream signalling pathways, such as the PI3K-AKT pathway, and antibody-dependent cellular cytotoxicity (ADCC), which also includes the inhibition of HER2 receptor dimerisation, receptor internalisation, and disruption.^30–32^ PARP inhibitors like olaparib exploit synthetic lethality with BRCA mutations by disrupting DNA repair in cancer cells, specifically targeting BRCA mutant cancer cells while sparing healthy ones.^33^ Bevacizumab (Avastin®), the first approved angiogenesis-targeting monoclonal antibody, has significantly impacted cancer therapy. It operates by inhibiting vascular endothelial growth factor A (VEGF-A), a key player in angiogenesis, thereby blocking VEGF signalling pathways from being activated. This has made it a central component in the treatment of angiogenesis-driven solid tumors like triple-negative breast cancer (TNBC) and HER2-negative breast cancer.^34^

There are significant studies showcasing the effectiveness of targeted drug delivery systems. Chowdhury *et al.* demonstrated that aptamer functionalised liposome substantially reduces the dose of doxorubicin and improves the therapeutic benefits by promoting the targeted delivery to the Her2-positive breast cancer cells.^35^ An investigation carried out by Ghosh *et al.* in 2021 demonstrated that the targeted transport of curcumin using hyaluronic acid-modified mesoporous silica nanohybrids triggers cancer cell death through mechanisms involving the generation of reactive oxygen species (ROS), cell cycle arrest, and the modulation of both NF-κB and the Bax-mediated apoptotic pathway.^36^ Additionally, Cao *et al.* conducted research in 2023, where they delivered doxorubicin *via* MTX-PEG-modified CG/DMMA polymeric micelles to target triple-negative breast cancer. This approach induced autophagy and displayed enhanced anti-tumor efficacy.^37^

These treatments focus on the molecular mechanisms and receptors involved in the development and spread of breast cancer. The correlation between molecular mechanisms and treatment in breast cancer involves identifying genetic mutations, signaling pathways, and gene expressions that influence tumor development.^38^ Targeted therapies directed at these molecular targets, like HER2 and PI3K/AKT pathways, aim to disrupt crucial oncogenic pathways and improve treatment effectiveness.^39^ Tailoring treatments based on the tumor's molecular subtype allows for optimized therapeutic outcomes.^40^ Together with targeted drug delivery platforms, they could result in highly focused and efficient tumor treatment.^11^ Overall, targeted drug delivery represents a promising avenue for breast cancer therapy, offering the potential for increased treatment efficacy, reduced side effects, and improved patient outcomes. Ongoing research and development in this field, supported by studies on nanocarrier design, targeted therapy mechanisms, and clinical trials, continue to advance our understanding and application of this innovative approach in the breast cancer treatment.^25^

Scientific advancements, as evidenced by molecular characterisation, targeted therapies, and innovative drug delivery systems, continue to shape the landscape of breast cancer research and treatment. Ongoing efforts to improve early detection, unravel the complexities of tumor heterogeneity, and develop more precise and effective therapies hold great promise for the future, ultimately aiming to enhance patient care and outcomes. By examining the current state of knowledge and recent developments, this review addresses the dynamic landscape of breast cancer research and its critical implications for improving patient care and outcomes.

## 3. Approaches of targeted drug delivery

Targeted drug delivery has surfaced as a cornerstone in disease therapy, reflecting the quest for more precise and effective treatment approaches. At its core, it seeks to optimise the therapeutic impact of anticancer agents while minimising their toxic effects on healthy tissues. In the context of breast cancer, this approach relies on a deep understanding of the molecular targets and biomarkers intimately associated with the disease.^27^ Breast cancer is not a singular entity but a complex group of diseases, each characterised by distinct molecular profiles. This heterogeneity poses a challenge for treatment because patient response to therapies can vary significantly. Consequently, identifying specific molecular targets and biomarkers becomes crucial for tailoring treatment strategies.^3^

### 3.1. Molecular targets and biomarkers

Molecular targets and biomarkers have revolutionised the field of breast cancer management. These include hormone receptors, such as estrogen receptor (ER) and progesterone receptor (PR), whose presence or absence informs the choice of hormonal therapy.^41^ HER2 is another pivotal biomarker, which has led to the development of HER2-targeted therapies like Herceptin, dramatically improving outcomes for HER2-positive breast cancer patients.^42^

**Fig. 2:**
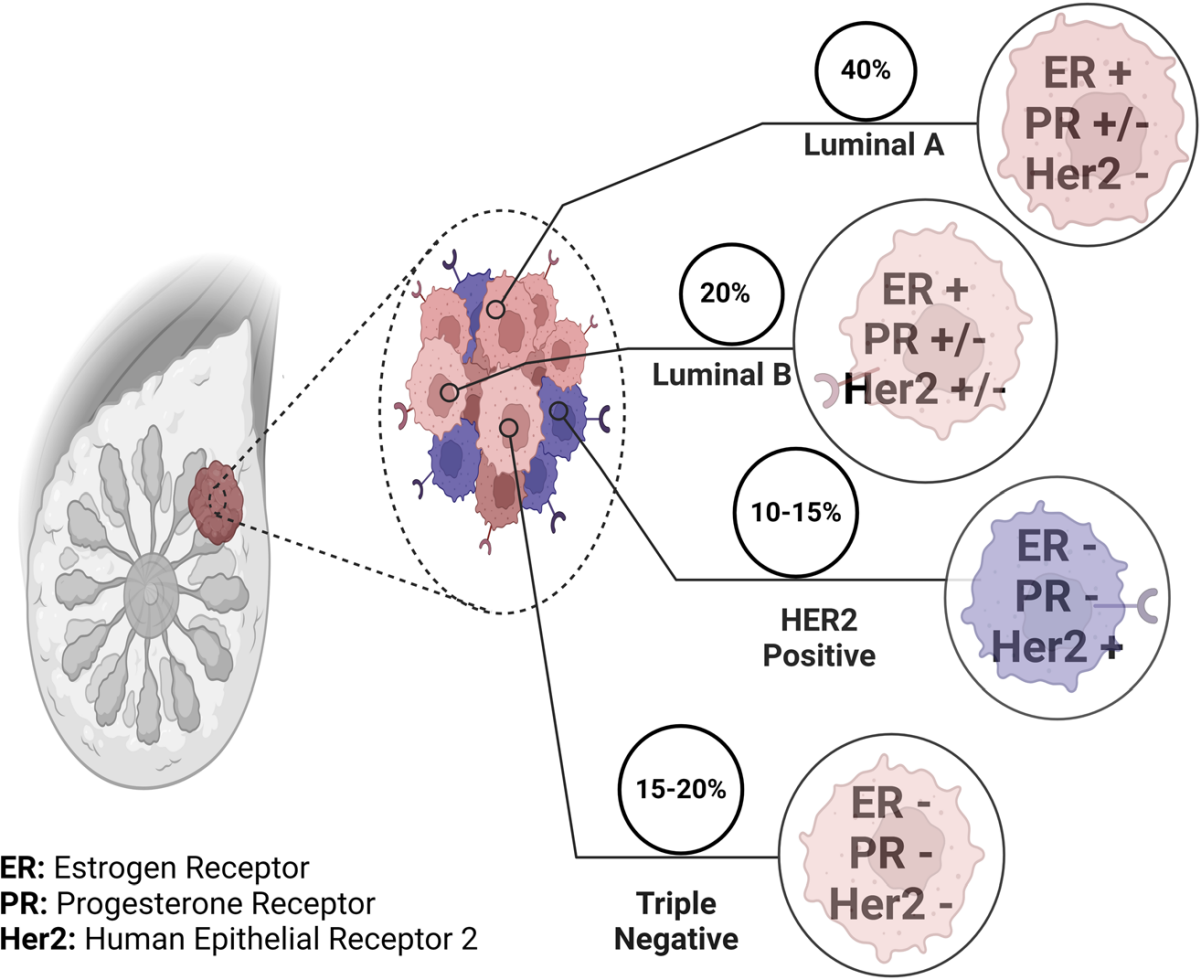
Molecular subtypes of breast cancer. Breast cancer is classified into four major molecular subtypes based on the expression of specific receptors, including estrogen receptor (ER), progesterone receptor (PR), Human Epidermal Growth Receptor 2 (HER2), and the absence of all three receptors (triple negative). Luminal A subtype is the most prevalent, accounting for approximately 40% of all cases, followed by luminal B (20%), HER2 positive (10–15%), and triple negative (15–20%). Understanding the prevalence of these subtypes is critical for tailoring treatment approaches and improving patient outcomes. This figure has been created with https://www.biorender.com, copyright 2024.

Furthermore, advancements in genomics have unveiled the intricate molecular subtypes of breast cancer, such as luminal A, luminal B, HER2-enriched, and triple-negative breast cancer (Fig. 2).^43^ Each subtype exhibits unique genetic signatures, clinical behaviours, and therapeutic responses. Biomarkers associated with these subtypes guide treatment decisions, ensuring a more tailored and effective approach.^3,43^

Emerging biomarkers, such as BRCA1 and BRCA2 mutations, indicate breast cancer risk and inform preventive strategies and therapeutic choices.^44^ Comprehensive molecular profiling has the potential to uncover additional targets and refine patient stratification, facilitating personalised medicine approaches in breast cancer management.^45^

### 3.2. Targeting strategies for breast cancer

Targeted drug delivery systems have revolutionised the treatment landscape for breast cancer, offering the potential for greater therapeutic efficacy while minimising systemic toxicity.^46^ This can be engineered to overcome drug resistance mechanisms that often hinder treatment success in breast cancer.^47^ By enhancing drug delivery to resistant cancer cells or employing combination therapies that address multiple resistance pathways, targeted drug delivery holds excellent potential in circumventing drug resistance.^11^ This section delves into various targeting strategies to enhance drug delivery, specifically to breast cancer cells, highlighting recent advancements and their clinical implications.

### 3.3. Passive targeting through enhanced permeability and retention (EPR) effect

**Fig. 3:**
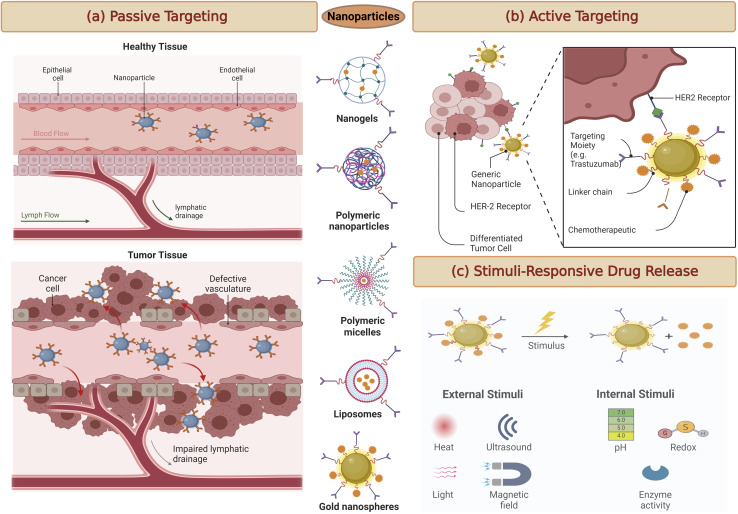
A schematic representation highlighting three distinct approaches in drug delivery systems. (a) Enhanced Permeation and Retention (EPR), (b) active targeting with ligands or antibodies and (c) stimuli-responsive drug release. This figure has been created with https://www.biorender.com, copyright 2024.

Targeting passively in breast cancer therapy relies on a fundamental phenomenon known as the enhanced permeability and retention (EPR) effect (Fig. 3a). This effect exploits the distinct characteristics of the tumor microenvironment.^48^ In many solid tumors, including breast cancer, the blood vessels supplying nutrients to the tumor are often abnormal. They are leaky and irregularly shaped, allowing nanoparticles and drug carriers to passively enter the tumor tissue.^49^

Once inside the tumor, these nanoparticles tend to accumulate due to the poor lymphatic drainage within the tumor. The lymphatic system, responsible for clearing fluid and waste products from tissues, is often compromised in cancer, further contributing to the retention of nanoparticles within the tumor microenvironment.^50^

Recent research has focused on optimising the design of nanoparticles and drug carriers to maximise the EPR effect. Parameters such as particle size, surface charge, and drug release profiles are carefully tailored to enhance drug delivery to breast tumors while minimising off-target effects.^51^ By exploiting the EPR effect, researchers aim to improve the selectivity and efficacy of breast cancer treatment.

### 3.4. Active targeting using ligands and antibodies

Active targeting strategies for breast cancer therapy take a more precise approach by utilising specific molecules, such as ligands, antibodies, or peptides, to guide drug carriers to their intended target cancer cells actively (Fig. 3b).^52^ These targeting moieties are selected based on their ability to bind with high affinity to receptors often overexpressed on cancer cell's surfaces.^53^ By conjugating these targeting ligands to drug carriers, such as nanoparticles or liposomes, drug delivery can be directed precisely to the tumor site. This approach significantly reduces off-target effects, sparing healthy tissues from the harmful effects of chemotherapy or other therapeutic agents.^54^

One of the remarkable advancements in this field is the development of antibody–drug conjugates (ADCs) for breast cancer treatment. ADCs consist of monoclonal antibodies that specifically recognise cancer cell surface receptors linked to potent cytotoxic payloads. This combination allows for a highly specific and potent therapeutic approach, where the antibody delivers the cytotoxic drug directly to the cancer cell, resulting in cell death while sparing normal cells.^55^

### 3.5. Stimuli-responsive drug delivery systems for breast cancer

Stimuli-responsive drug delivery systems represent a cutting-edge approach to breast cancer therapy. These systems are designed to release therapeutic agents only when triggered by specific conditions within the tumor microenvironment (Fig. 3c).^56^ Such conditions may include variations in pH, temperature, or enzymatic activity unique to cancer cells. Stimuli-responsive drug carriers are engineered to respond to these cues, ensuring precise drug release within the tumor while sparing healthy tissues. For example, in breast cancer, the slightly acidic environment of tumors can be exploited as a trigger for drug release.^57^ As nanoparticles or carriers enter the tumor tissue, the acidic conditions prompt them to release the therapeutic payload, maximising drug exposure to cancer cells and minimising side effects in surrounding tissues.^58^

This approach offers several advantages, including improved drug bioavailability at the target site and reduced systemic toxicity. It holds great promise for enhancing therapeutic efficacy in breast cancer treatment.

## 4. Types of targeted drug delivery systems in breast cancer

**Fig. 4:**
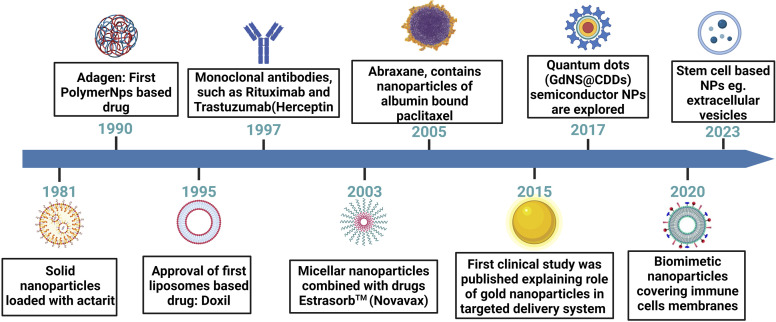
A schematic presentation of a chronological journey through the evolution of drug delivery systems. This figure has been created with https://www.biorender.com, copyright 2024.

The quest for more effective and precise cancer therapies has led to the emergence of various novel targeted drug delivery systems. These innovative approaches are designed to enhance the delivery to malignant cells while sparing the healthy tissues. This section explores the diverse landscape of targeted delivery platforms for cancer, such as nanoparticle-based systems, liposomal formulations, ADCs, and polymer-based carriers (Fig. 4). Each of these systems harnesses distinct technologies and mechanisms to enhance the therapeutic impact of anti-cancer drugs. Through an in-depth examination of these approaches, we aim to highlight the evolving strategies that hold promise in the battle against cancer, with a particular focus on breast cancer.

### 4.1. Nanoparticle-based drug delivery systems

Utilising nanoparticles in targeted drug delivery for cancer therapy offers a multitude of advantages. These include precise targeting through functionalisation with ligands, enhanced solubility of hydrophobic drugs, and controlled release profiles optimising therapeutic outcomes.^59^ Nanoparticles also afford protection to encapsulated drugs, exhibit biocompatibility, and can traverse biological barriers for improved drug delivery to specific tissues.^60^ Furthermore, their multi-functionality allows for personalised approaches in diagnostics and treatment monitoring. At the same time, high cellular uptake rates ensure efficient drug delivery to target cells, collectively enhancing the efficacy and versatility of nanoparticle-based drug delivery systems in cancer treatment.^61^

Recent advancements in nanoparticle-mediated delivery platforms have shown ground-breaking developments in breast cancer treatment. For instance, researchers have harnessed the potential of albumin nanoparticles loaded with paclitaxel (Abraxane®).^62^ This formulation improves the solubility of paclitaxel, enhancing its delivery to breast cancer cells and allowing for lower doses and reduced side effects.^63^ More recently, a study involving breast cancer patients demonstrated the enhanced efficacy of Abraxane® compared to conventional paclitaxel formulations.^64,65^

The Au^+^ ions with a positive charge within gold nanoparticles (GNPs) play a pivotal role in their function as carriers that specifically target tumors. GNPs attract negatively charged biomolecules *via* electrostatic interactions. Simultaneously, GNPs are modified with organic or polymeric ligands through linker molecules that contain thiol or nitrogen (N) groups. This connection results in a range of diverse physiological effects.^66,67^ As an illustration, in a study by Li and colleagues, a unique approach was employed to create yolk–shell nanoparticles based on calcium phosphate (CaP). These nanoparticles featured a removable gold nanorod yolk. Notably, these nanoparticles exhibited exceptional loading efficiency, with the capacity to encapsulate doxorubicin (DOX) molecules reaching up to 100%. Furthermore, they demonstrated a dual-response mechanism sensitive to pH and near-infrared (NIR) light, enabling them to accumulate within tumors and release DOX when exposed to either acidic conditions or NIR laser stimulation.^68^

Magnetic nanoparticles (MNPs) have also been explored as they possess a unique superparamagnetic quality within their crystalline core. This feature allows for altering their microwave magnetic response when exposed to an external polarised magnetic field, without causing any disruption to the surrounding environment. As a result, they are important contrast agents in the realm of cancer diagnosis and tumor imaging. Additionally, the selectivity of MNPs in targeting tumor tissues, using specific antigens present on tumor cell receptors, positions them as promising vehicles for targeted drug delivery in research.^69^ Zou and colleagues^70^ synthesised mesoporous MNPs with DOX encapsulated and functionalised with chitosan to enhance their stability and circulation. These mesoporous MNPs exhibited a substantial DOX encapsulation and demonstrated the efficiency of targeting breast cancer when subjected to alternating current (AC) electromagnetic fields. Furthermore, in another investigation by Semkina *et al.*, polyethylene-glycolized magnetic NPs (PEG-MNPs) were functionalised with anti-vascular endothelial growth factor (VEGF) monoclonal antibodies for the delivery of DOX. This approach facilitated the accumulation of these PEG-MNPs at the tumor and the magnetic core generated robust signals that were detectable by real-time magnetic resonance imaging (MRI) monitoring.^71^ Recently, Hu *et al.* formulated hyaluronic acid-modified hollow copper sulfide nanoparticles encapsulating diethyldithiocarbamate (DDTC), combined with losartan, to enhance photothermal therapy (PTT) for breast cancer. This approach improves drug accumulation, enhances anti-tumor effects, induces effective immunogenic cell death (ICD), and remodels the tumor microenvironment, inhibiting metastatic tumor development.^72^

### 4.2. Liposomal formulations for targeted delivery

Liposomes represent a versatile targeted drug delivery system with numerous advantages. They shield drugs from degradation, minimize side effects by reducing exposure to healthy tissues, and enhance drug stability.^73^ Tailored modifications enable precise delivery to specific cells or tissues, improving treatment accuracy and efficacy.^74^ Additionally, liposomes offer customizable characteristics, including composition and size, and are biocompatible and biodegradable, ensuring safety for drug delivery applications.^75^ Leveraging these attributes, liposomes hold substantial potential to enhance the effectiveness and safety of drug treatments across various medical fields.^76^

Liposomes are spherical structures consisting of biodegradable and biocompatible lipid bilayers. These lipid bilayers provide a unique environment, enabling the encapsulation of hydrophilic drugs within the aqueous core, while simultaneously safeguarding hydrophobic drugs within the lipid membrane.^30^ Liposomal formulations have been significantly refined to enable precise targeted delivery in breast cancer therapy. Recent developments include using liposomes conjugated with monoclonal antibodies to target overexpressed receptors on cancer cells. Notably, liposomal doxorubicin coupled with trastuzumab (Herceptin®), an anti-HER2 antibody, enhances drug delivery to HER2-positive breast cancer cells, improving therapeutic outcomes.^77^ Liang and colleagues^78^ utilized cationic liposomes decorated with peptide-p37 (CDO14) to administer siRNA in breast cancer cells overexpressing heat-shock-protein-gp96. The p37 peptide, known for inhibiting gp96, a novel tumor therapy target, was introduced to enhance liposome targeting. Their experiments revealed a remarkable gene silencing efficacy with p37-CDO14, and significantly higher tumor inhibition efficacy than unmodified liposomes. In a study by another research team, they employed thermosensitive liposomes containing the photosensitiser cyanine dye and the anti-cancer natural plant compound (parthenolide) for a combinatorial approach to treat TNBC.^79^ When exposed to NIR light, indocyanine green generated heat, causing a transformation in the structure of the thermosensitive liposomes, leading to the release of the drug. This specialised liposomal formulation exhibited a 2.08-fold increase in tumor suppression compared to paclitaxel. However, it is essential to note that further investigation and *in vivo* validation are necessary to support these findings.^79^ Jain *et al.* have pioneered the development of pH-responsive liposomes, loaded with DTX and surface-functionalized with VEGF antibodies, aimed at optimizing breast cancer treatment. Their investigation revealed heightened cellular uptake, an enhanced drug release profile under acidic microenvironments, and an extended pharmacokinetic half-life when compared to free DTX.^80^ In a complementary study, Cao *et al.* targeted the powerful anticancer agent emtansine using a biomimetic drug delivery method utilising pH-sensitive liposomes covered with macrophage membranes. This approach enhances liposomes' capacity for specific metastatic site targeting. Their findings substantiate the notable improvement in the specificity of lung metastasis targeting in breast cancer, resulting in substantial growth inhibition. These innovative strategies hold significant potential for advancing breast cancer therapy.^81^

### 4.3. Antibody–drug conjugates (ADCs)

Antibody–drug conjugates in targeted drug delivery offer precision targeting to cancer cells, minimising off-target effects and systemic toxicity. They enhance treatment efficacy by concentrating cytotoxic drugs at tumor sites and can overcome biological barriers. ADCs can be combined with other therapies for synergistic effects and tailored to specific tumor types for personalised medicine. Overall, ADCs present a promising strategy to improve the efficacy and safety of cancer treatments.^82^

ADCs have recently emerged as a potent class of targeted therapies for breast cancer. ADCs represent a novel class of biopharmaceuticals that comprise mAbs chemically linked to small-molecule drugs through bioactive connectors. Throughout their developmental journey, two critical determinants affecting the efficacy of ADCs have become increasingly apparent: the meticulous design of the linker connecting the mAb to the therapeutic payload and the strategic conjugation of a potent chemotherapeutic agent to the monoclonal antibody.^83^

The pioneering ADC, Gemtuzumab ozogamicin (Mylotrag®), was the first of its kind and received approval in 2000. This ADC comprises Gemtuzumab, conjugated to *N*-acetyl gamma calicheamicin dimethyl hydrazide through non-specific lysine attachment. Notably, its linker featured a hydrazone bond designed to be cleaved within the acidic intracellular environment of target cells, thereby releasing the anti-tumor antibiotic calicheamicin. However, it was observed that this ADC's linker was prone to instability in the bloodstream, resulting in the premature release of cytotoxic calicheamicin payloads. This unexpected release led to unintended toxic effects, prompting Pfizer to withdraw Gemtuzumab ozogamicin from the market in 2010 voluntarily.^84^

Sacituzumab govitecan-hziy (Trodelvy®), an ADC targeting Trop-2-expressing breast cancer, has demonstrated remarkable clinical efficacy and received FDA approval.^62^ Recent clinical trials have further supported the effectiveness of Trodelvy® in refractory metastatic triple-negative breast cancer,^64,85^ highlighting the pivotal role of ADCs in modern breast cancer treatment.

### 4.4. Polymer-based drug carriers

Polymeric nanoparticles are favoured in targeted drug delivery due to their numerous advantages. They offer controlled drug release, minimising dosing frequency and allowing sustained release profiles. Functionalisation enables precise targeting, reducing off-target effects and enhancing therapeutic outcomes.^86^ Additionally, polymeric nanoparticles improve pharmacokinetics by increasing drug accumulation at target sites while reducing systemic toxicity.^87^ They can encapsulate a wide range of drugs, including hydrophobic and hydrophilic compounds, proteins, genes, and imaging agents, for versatile applications.^88^ Moreover, their biocompatibility, ease of functionalisation, scalability, and reproducibility make them promising for clinical translation. These attributes highlight the potential of polymeric nanoparticles as effective drug delivery systems across diverse therapeutic applications.^89^

Innovations in polymer-based drug carriers are driving personalised breast cancer treatment strategies. Polymeric micelles, designed for controlled drug release and improved bioavailability, represent a recent breakthrough. Recent research has focused on dual pH-responsive micelles for co-delivery of axitinib and paclitaxel, enhancing their anticancer efficacy against breast cancer.^90^

Peng *et al.* developed engineered worm-like nanocrystal micelles by conjugating Herceptin with PCL–PEG for the targeted treatment of HER2^+^ overexpressing breast cancer. These micelles, containing paclitaxel (PTX) and Herceptin, exhibited remarkable stability within the bloodstream and the tumor microenvironment (TME), precisely targeting HER2^+^ positive cells.^91^ Concurrently, Garg *et al.*, devised traceable polymeric micelles termed PEO–poly(α-benzyl carboxylate-ε-caprolactone) (PEO–PBCL) by incorporating pendant benzyl carboxylate groups into the PCL segment of PEO–PCL. Additionally, they integrated the NIR probe Cy5.5 into the core-forming block of these micelles. As a result, these modified micelles displayed superior accumulation at tumor sites, increased stability, and the ability to monitor disease progression in real-time within *in situ* breast cancer mouse models. These advancements hold substantial promise for precise and effective breast cancer therapy.^92^ In the study by Aleanizy and colleagues, they devised a delivery system using a PAMAM dendrimer combined with trastuzumab to administer an adjuvant. Their findings highlighted the enhanced selectivity, cytotoxicity, and increased cellular uptake of these dendrimers when compared to the standalone drugs. These outcomes suggest that these dendrimer-based systems hold considerable promise as targeted drug delivery platforms for breast cancer treatment.^93^

### 4.5. Other emerging targeted delivery approaches

**Fig. 5:**
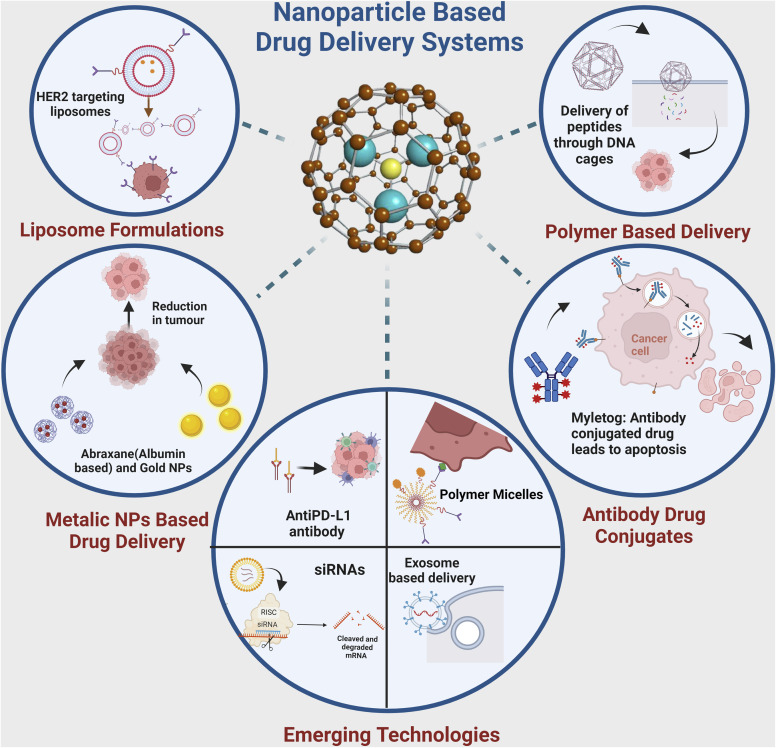
Emerging targeted drug delivery systems for precision breast cancer therapy. Nanoparticles, liposomes, antibody–drug conjugates, polymeric micelles, and exosomes can be engineered to selectively deliver chemotherapeutic agents, RNA therapeutics, and immunotherapy to breast tumors. Surface functionalisation with tumor-targeting ligands enhances selective uptake. Stimuli-responsive and multifunctional designs allow controlled drug release and real-time monitoring. These innovative technologies aim to improve treatment efficacy and safety through targeted delivery to cancer cells while sparing healthy tissues. This figure has been created with https://www.biorender.com, copyright 2024.

In addition to nanoparticle-based systems, liposomal formulations, ADCs, and polymer-based carriers, several innovative targeted delivery approaches are emerging in breast cancer treatment presented in Fig. 5. These approaches leverage advanced technologies and biological insights to enhance the precision and efficacy of therapies. Following are some noteworthy emerging strategies:

#### 4.5.1. Exosome-based drug delivery

Exosomes are small vesicles secreted by cells that play a crucial role in intercellular communication.^94^ Recent research has explored the use of exosomes as natural drug delivery carriers. Exosomes offer targeted drug delivery advantages in cancer therapy, boasting surface proteins for precise cell targeting and a lipid bilayer structure protecting cargo from degradation.^95^ Their role in intercellular communication aids in modulating cellular responses, while prolonged circulation ensures sustained drug release and improved bioavailability. With low immunogenicity and the ability to cross biological barriers, exosomes stand as a promising platform for enhancing efficacy and safety in cancer treatment.^96^ This approach shows promise in harnessing the body's communication system for drug delivery. Bagheri *et al.* engineered a DOX-loaded exosome from MSCs, achieving 35% encapsulation efficiency.^97^ Functionalisation with the MUC1 aptamer improved cancer cell targeting. In a C26 carcinoma mouse model, MUC1apt–MExo–DOX significantly reduced cancer volume and ensured 100% survival after 30 days, demonstrating its efficacy in cancer therapy.^98,99^ Yong *et al.* introduced an inorganic NP-exosome hybrid structure for delivering anti-cancer agents, wherein DOX was loaded into mesoporous silica NPs (DOX–MPS) and applied to cancer cells. The DOX–MPSs entered H22 or Bel7402 cells *via* endocytosis, forming DOX–MPS/exosome core/shell structures. These hybrids exhibited anti-cancer effects in H22 tumor-bearing mice and B16–F10 lung metastasis mice, confirming their therapeutic potential.^100^

#### 4.5.2. Peptide-targeted therapies

Peptides are short chains of amino acids designed to bind specifically to surface receptors on cancer cells. Peptide-based targeted therapies are gaining traction for breast cancer treatment. These peptides can serve as homing devices for drug-loaded nanoparticles or as components of ADCs, ensuring precise drug delivery to tumor cells and avoiding healthy tissues.^101,102^ This method holds the potential for personalised treatment based on specific molecular characteristics of the breast tumor.^74^

Du *et al.* developed PEG-conjugated peptides DH6 (YLFFVFER) and RDH6 (REFVFFLY), demonstrating specific targeting of HER2-positive tumors with good metabolic stability.^103^ Stefanick *et al.* also investigated HER2-targeting peptides HERP5, HRAP, KAAYSL, and AHNP for cellular uptake, with KAAYSL exhibiting the highest tumor uptake.^104^ Hailing *et al.* developed nanoparticles composed of GE11-modified polylactic-*co*-glycolic acid (PLGA) and d-α-tocopheryl polyethylene glycol 1000 succinate to deliver salinomycin specifically to breast cancer cells. Their findings demonstrated that these nanoparticles significantly improved therapy efficacy, particularly in breast cancer cases with overexpression of EGFR.^105,106^

#### 4.5.3. RNA-based therapeutics

Advances in RNA-based therapeutics, including small interfering RNA (siRNA) and messenger RNA (mRNA), are transforming breast cancer treatment. These molecules can be designed to target specific genes or proteins involved in cancer growth and progression. Nanoparticle-based carriers can deliver high-precision RNA therapeutics to breast cancer cells, offering a potential avenue for gene silencing or protein expression modulation.^107^

Yan *et al.* engineered nanosized liposomes conjugated with tLyp-1 peptide to target neuropilin (NPR) receptors on breast cancer (BC) tumor cells. This nanoformulation, loaded with a miR-203 mimic, induced post-transcriptional silencing of Slug and suppressed the TGF-β1/Smad pathway both *in vitro* and *in vivo*.^108^ A targeted delivery system was developed using poly(β-amino ester) and poly(d,l-lactide-*co*-glycolide) polymers, delivering antimir-21 and epirubicin to cancer cells. MUC1 aptamer modification facilitated specific uptake by MCF7 and C26 cells, reducing viability without affecting MUC1-negative CHO cells. This nanocomplex showed enhanced efficacy and safety in reducing tumor growth in mouse models compared to epirubicin alone.^109^ Nayak *et al.* developed cationic liposomes comprising dicta decyl amido glycyl spermidine (DOGS) and DOPE to transport siRNAs to breast cancer cell lines, demonstrating successful delivery and specific localization near the nucleus. These liposomes exhibited low cytotoxicity and facilitated high uptake of cyclin D1-specific siRNA in MCF-7 cells, along with efficient delivery of plasminogen activator inhibitor type I-specific siRNA to MDA MB 231 cells.^110^

#### 4.5.4. Immune checkpoint inhibitor delivery

Immunotherapy is a promising avenue in breast cancer treatment, particularly in aggressive subtypes. Targeted delivery of immune checkpoint inhibitors, such as anti-PD-L1 antibodies, to the tumor microenvironment is being explored. This approach aims to enhance the anti-tumor immune response while minimising systemic side effects, potentially improving the efficacy of immunotherapy in breast cancer.^30^

Bakhos *et al.* utilized virus-like particles (VLPs) to deliver the STING agonist 2′3′-cGAMP.^111^ 2′3′-cGAMP, a natural mammalian STING agonist, activates STING in DCs immediately upon fusion, packaged within enveloped virus particles.^112,113^ The synthesized VLPs encapsulating cGAMP consist of HIV-1 structural protein and vesicular stomatitis virus glycoprotein envelope glycoprotein. This study demonstrated that cGAMP-VLPs were approximately fifty times more efficient than conventional liposomes in delivering cGAMP into cells.^114^

## 5. Targeted drug delivery at preclinical and clinical level

In targeted drug delivery for breast cancer, the journey from conception to clinical application involves a meticulous exploration encompassing preclinical studies in laboratory models and rigorous clinical trials involving breast cancer patients. This section provides a comprehensive overview of preclinical investigations and clinical trials, offering insights into the progress, challenges, and notable outcomes in targeted drug delivery for breast cancer.

### 5.1. Overview of preclinical studies on targeted drug delivery in breast cancer models

Preclinical studies are the foundational cornerstone in assessing the efficacy and safety of delivery systems. These studies predominantly employ laboratory models, including cell cultures and animal models, to replicate and comprehend the behaviour of drug carriers and therapeutic agents within the intricate tumor microenvironment.^29^ Preclinical investigations serve multifaceted purposes. Researchers employ these studies to delve into critical aspects, encompassing drug carriers' pharmacokinetics, biodistribution, and toxicity profiles. Furthermore, they meticulously scrutinise the capacity of these systems to precisely and effectively target breast cancer cells while minimising detrimental effects on healthy tissues. Preclinical research often involves fine-tuning carrier attributes, encompassing particle size, surface chemistry, drug release kinetics, and the integration of targeting moieties to optimise therapeutic efficacy.^58^

Recent preclinical studies have unveiled the potential of a diverse array of targeted drug delivery approaches. These investigations have illuminated enhanced drug accumulation within breast tumors, amplified anti-tumor effects, and mitigated systemic side effects.^115^ These findings, rooted in scientific rigour, are pivotal stepping stones, paving the way for subsequent clinical translation.

### 5.2. Clinical trials and outcomes of targeted drug delivery systems in breast cancer patients

Clinical trials are pivotal in evaluating targeted drug delivery systems for breast cancer treatment. These trials pivotally involve breast cancer patients and are meticulously designed to gauge these innovative therapeutic paradigms' safety, efficacy, and overall clinical benefits.^55^

Recent clinical trials have traversed a comprehensive spectrum of targeted drug delivery systems, encompassing nanoparticle-based formulations, ADCs, and stimuli-responsive carriers, evaluated across diverse breast cancer subtypes. These trials invariably focus on specific patient cohorts, such as those afflicted with HER2-positive or triple-negative breast cancer, and methodically scrutinise endpoints spanning tumor response rates, progression-free survival, and the holistic impact on patient's quality of life.^116^

Promising outcomes from recent clinical trials have not only led to the approval of several targeted drug delivery systems for breast cancer treatment but have also rekindled hope among patients. Notable examples include the HER2CLIMB trial, which demonstrated the efficacy of tucatinib in HER2-positive breast cancer, and the ASCENT trial, showcasing the benefits of Sacituzumab govitecan in metastatic triple-negative breast cancer.^77,117^ Nevertheless, challenges, such as patient selection criteria, optimal dosing strategies, and the emergence of resistance mechanisms, remain subjects of ongoing research and refinement.^118^

## 6. Challenges and future directions

The quest for optimised targeted drug delivery in breast cancer faces formidable challenges, necessitating a concerted effort to address these hurdles. This section scrutinises these challenges through a scientific lens and explores potential future directions guided by empirical evidence and research findings.

### 6.1. Overcoming biological barriers in targeted drug delivery to breast cancer

**Fig. 6:**
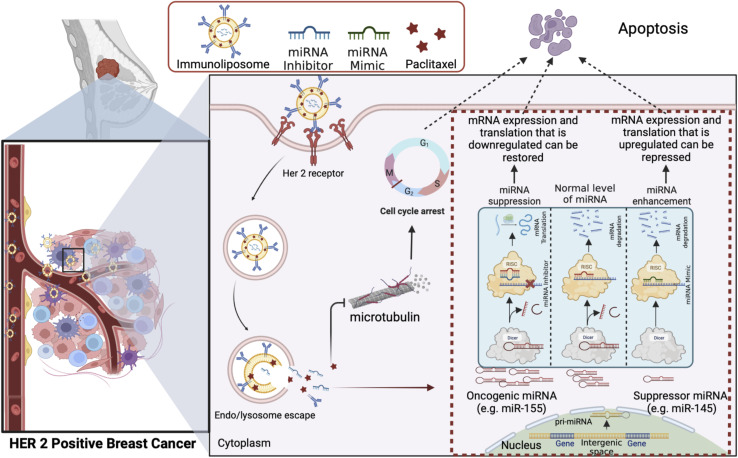
Next-generation liposomes: immunoliposomes, armed with anti-HER2 antibodies, encapsulate conventional chemotherapeutic drugs and specific miRNAs linked to drug resistance mechanisms. This precise dual-targeted approach ensures the selective delivery of therapeutic cargo to cancer cells, sensitising them to chemotherapy through miRNA modulation *via* miRNA inhibitors or mimics. The combined delivery of miRNAs and drugs results in a potent synergistic effect, allowing for lower drug concentrations while inducing apoptosis in cancer cells, potentially revolutionising the treatment of HER2-positive breast cancer by overcoming drug resistance and minimising adverse effects. This figure has been created with https://www.biorender.com, copyright 2024.

The intricate tumor microenvironment is a dynamic and heterogeneous landscape, imposing formidable barriers to precise drug delivery. Achieving optimal drug penetration, circulation stability, and specificity is an ongoing challenge.^119^ Strategies to surmount these challenges are pivotal for advancing the field. Fig. 6 represents one promising approach to overcoming drug resistance by exploiting the combined delivery of a chemotherapeutic drug and the microRNA associated with its drug resistance *via* an immunoliposome based delivery system to target the cancer cells while sparing the healthy tissues.

Recent strides in nanoparticle engineering offer encouraging prospects. For instance, Zheng *et al.* (2020) have devised pH-responsive nanoparticles that adeptly navigate the intricacies of the tumor microenvironment, facilitating controlled drug release and significantly improving therapeutic efficacy.^119^

### 6.2. Biodegradation and clearance of nanocarriers

In the realm of nanomedicines, understanding the biodegradation and clearance of drug carriers post-delivery is paramount for assessing their safety profile and potential long-term effects.^120^ The breakdown of nanocarriers is pivotal, influencing the release of encapsulated drugs and metabolites within the body.^121^ Additionally, clearance pathways, whether renal excretion, hepatic metabolism, or other routes, are critical for evaluating systemic impact and drug pharmacokinetics.^122^

Recent investigations have elucidated the biodegradation and clearance pathways of diverse nanocarrier systems utilised in drug delivery. For instance, studies on polymeric nanoparticles, such as PLGA (poly(lactic-*co*-glycolic acid)) carriers, have revealed their ability to biodegrade into harmless metabolites that are eventually excreted from the body.^123^ Similarly, research on lipid-based nanocarriers, like liposomes, has demonstrated degradation processes that enable the safe elimination of both the carrier and the loaded drug.^124^

Moreover, the exploration of hybrid nanocarriers, which combine different materials such as polymers and metals, has provided insights into their biodegradation patterns and clearance mechanisms.^120^ Investigations into silica-based nanocarriers have highlighted their potential for controlled degradation and safe elimination from the body.^125^ Additionally, the utilisation of biodegradable dendrimers as nanocarriers has shown potential in facilitating effective drug release while ensuring biocompatibility and eventual clearance.^126^

### 6.3. Immunological considerations and strategies for improved efficacy

Immunological nuances play a pivotal role in targeted drug delivery to breast cancer. The interplay between drug carriers, therapeutic agents, and the host immune system significantly influences treatment outcomes.^127^ Strategies to harness and modulate these interactions hold immense potential.

Recent research by Gu *et al.* (2022) underscores the importance of incorporating immunomodulatory agents into targeted drug delivery paradigms. Their investigation into the co-delivery of immunostimulatory compounds alongside chemotherapy has yielded profound insights, resulting in enhanced tumor regression and robust immune response in breast cancer models.^128^

### 6.4. Translational challenges

Despite the promising potential of nanomedicines in targeted drug delivery for breast cancer therapy, their clinical translation faces significant hurdles.^129^ The complexity of multicomponent nanosystems predisposes them to instability, aggregation, and uncontrolled drug release, raising concerns about potential toxicities from the nanocarriers or the formation of a protein corona in biological fluids.^130^ Scaling up production while maintaining desired physicochemical attributes and therapeutic efficacy presents a daunting task. *In vivo*, these nanomedicines encounter biological barriers such as the dense extracellular matrix and elevated interstitial fluid pressure in the tumor microenvironment, as well as physiological challenges like the blood–brain barrier in metastatic disease.^131^ The adsorption of plasma proteins onto nanoparticle surfaces, forming a protein corona, profoundly affects their biodistribution, cellular uptake, and toxicity profiles. Preclinical assessment is complicated by limitations in conventional animal models, necessitating the development of more representative platforms such as organoids.^132^ Furthermore, the absence of harmonised regulatory guidelines for evaluating the quality, safety, and efficacy of complex nanomedicines presents a significant obstacle to their successful clinical translation. Collaborative efforts across disciplines are essential to overcome these multifaceted challenges and advance the translation of targeted drug delivery systems for breast cancer therapy.^133,134^

### 6.5. Regulatory aspects and commercialisation prospects

Navigating the regulatory landscape and realising commercialisation prospects constitute critical milestones in translating targeted drug delivery systems for breast cancer. Regulatory approvals hinge on compelling preclinical and clinical evidence substantiating safety and efficacy.^135^ Commercial success demands considerations of scalability, cost-effectiveness, and market accessibility.

Recent regulatory greenlights and commercial victories underscore the immense potential of targeted drug delivery in breast cancer therapy. Notable examples include the approval of trastuzumab emtansine (T-DM1) and Sacituzumab govitecan (SG) for HER2-positive and triple-negative breast cancer, respectively.^55,77^ These milestones illuminate the path forward and reinforce the imperative of continued investment in research and development.

## 7. Conclusion

As we conclude this comprehensive exploration of targeted drug delivery in breast cancer, it is evident that this field has witnessed remarkable strides, offering substantial promise for improved patient outcomes. In this conclusion, we summarise key findings and advancements, underscoring the transformative potential of targeted drug delivery in breast cancer treatment. Furthermore, we discuss the promising perspectives that lay the foundation for the future of this dynamic domain.

### 7.1. Summary of key findings and advancements in targeted drug delivery for breast cancer

Recent years have seen significant progress in targeted drug delivery strategies for breast cancer, propelled by innovative research and technology. Noteworthy findings and advancements include:

#### 7.1.1. Precision and specificity

Targeted drug delivery systems have substantially enhanced the precision and specificity of therapeutic agents, minimising off-target effects. Notable examples include the use of trastuzumab emtansine (T-DM1) for HER2-positive breast cancer and SG for TNBC.^55,77^

#### 7.1.2. Overcoming barriers

Innovative strategies, such as pH-responsive nanoparticles and immunomodulatory agents, have emerged to overcome biological barriers within the tumor microenvironment. These advances have improved drug penetration and therapeutic outcomes.^128,136^

#### 7.1.3. Immunomodulation

Integrating immunostimulatory compounds into targeted drug delivery systems has demonstrated the potential to elicit robust immune responses. This has opened new avenues for synergistic treatment approaches, as exemplified by combining chemotherapy with immunotherapies.^137^

#### 7.1.4. Commercial success

Regulatory approvals and successful commercialisation of targeted drug delivery systems, such as T-DM1 and SG, have reinforced the feasibility and commercial potential of tailored breast cancer therapies.^55,77^

#### 7.1.5. Patient-centric care

The evolution of targeted drug delivery aligns with the paradigm of personalised medicine, promising treatments that are increasingly tailored to individual patient profiles, molecular subtypes, and disease stages.^138^

### 7.2. Perspectives on the future of targeted drug delivery in breast cancer treatment

The future of targeted drug delivery in breast cancer treatment is imbued with promise and potential. Several key perspectives and directions merit attention:

#### 7.2.1. Precision oncology

Integrating genomic and proteomic data with advanced machine learning algorithms is expected to develop increasingly precise and personalised targeted drug delivery systems.^139^ Recent initiatives such as the TAILORx trial have demonstrated the potential for gene expression profiling to guide treatment decisions and optimise outcomes.^140^

#### 7.2.2. Combination therapies

The synergy between targeted drug delivery and emerging immunotherapies holds immense potential. Ongoing research endeavours aim to unravel the intricate interplay between the tumor microenvironment and the immune system, facilitating the design of more effective combination therapies.^137^

#### 7.2.3. Biomarker discovery

Biomarker discovery remains a focal point for research, enabling the identification of novel molecular targets and the development of companion diagnostics.^141^ Recent studies have highlighted promising biomarkers like PD-L1 and TILs (Tumor-Infiltrating Lymphocytes) as indicators of immunotherapy response.^142^

#### 7.2.4. Regulatory harmonization

Achieving regulatory harmonisation across regions is pivotal for expediting the translation of innovative targeted drug delivery systems into clinical practice.^143^ Recent international efforts, such as the World Health Organization's initiative on harmonising global regulatory standards, signify a step in the right direction.^144^

In conclusion, targeted drug delivery in breast cancer has evolved from a conceptual framework to a tangible reality with the potential to revolutionise patient care. Recent advancements, exemplified by precision therapies, innovative drug carriers, and immunomodulatory approaches, offer substantial hope. The future holds exciting prospects for even more personalised, effective, and accessible breast cancer treatments, with a growing emphasis on integrating multidisciplinary research and a patient-centric approach.

## Author contributions

Ramesh Chaudhari: literature review, data analysis, writing – original draft preparation. Vishva Patel: writing – reviewing and editing. Ashutosh Kumar (corresponding author): conceptualization, supervision, writing – reviewing and editing.

## Conflicts of interest

There are no conflicts to declare.

## Supplementary Material
